# Accessing and educating female sex workers in Ukraine via a peer-driven intervention

**DOI:** 10.1186/1742-4690-9-S1-P118

**Published:** 2012-05-25

**Authors:** Oksana Matiyash, Pavlo Smyrnov, Robert Broadhead

**Affiliations:** 1 International HIV-AIDS Alliance-Ukraine, Kyiv, Ukraine

## Introduction

A peer-driven intervention (PDI) for female sex workers (FSWs) was implemented in harm reduction projects in two Ukrainian cities. The goal was to recruit and interview 500 FSWs in 6 months who had never received services before, and to measure how well FSW-recruiters educated FSWs in a body of fresh HIV prevention information. Recruiters were rewarded by earning tickets to win special prizes in a weekly lottery.

## Methods

A PDI relies on respondent educating and recruiting peers for services. All recruits also get to serve as peer-educator/recruiters. Each recruit’s score on an 8-item knowledge test, measuring how well the recruiter educated her, determined how many lottery tickets the recruiter earned. A weekly lottery was held each week offering prizes to 4 lucky winners.

## Results

The two PDIs in 6 months recruited 532 and 437 FSWs never seen before, 2-3 times more new FWSW-recruits than the number recruited 6 months prior to the PDIs’ start-up. Both projects held 23 lotteries with an average attendance of 8 FSWs. Very significant differences in levels of drug- and sex-related risk behaviors were found between FSW drug-users and non-users, heavily shaped by education, knowledge, and other social variables, suggesting more targeted types of intervention.

## Conclusion

The FSW-respondents were eager to serve as peer-educators/recruiters. The lottery proved to be a cost-saving and effective reward system that was highly motivating. The PDI offers harm reduction projects an entirely new model for accessing and educating FSWs, as well as a new method for targeting special sub-populations of FSWs.

